# The Impact of International Missions in Provision of Cardiac Services and Skill Transfer in Respect to Coronary Artery Bypass Grafting at Jakaya Kikwete Cardiac Institute - Tanzania

**DOI:** 10.21470/1678-9741-2024-0075

**Published:** 2025-04-28

**Authors:** Evarist T. M. Nyawawa, Henry A. Mayala, Peter R. Kisenge, Moses Byomuganyizi, Alex B. Joseph, Angela N. Muhozya, Ramadhan Khamis, Evans E. Nyawawa, Juma B. Nyangasa, Adelphina Ncheye, Alex Loth, Aubyn Marath

**Affiliations:** 1 Jakaya Kikwete Cardiac Institute, Dar es Salaam, United Republic of Tanzania; 2 Department of Cardiothoracic Surgery, Muhimbili University of Health and Allied Sciences, Dar es Salaam, United Republic of Tanzania; 3 Sekou Toure Regional Referral Hospital, Mwanza, United Republic of Tanzania; 4 Department of Cardiothoracic Surgery, Oregon Health and Science University, Portland, Oregon, United States of America

**Keywords:** Coronary Artery Disease, Cardiopulmonary Bypass, Constriction, Medical Missions, Demography

## Abstract

**Objective:**

To assess how efficient the local team attained skills are after several visits made by international missions in respect to number of coronary artery bypass grafting surgery performed and the overall patient outcome.

**Methods:**

This was a retrospective study that included all patients who were operated on at the center after being diagnosed with chronic coronary artery disease from May 2016 to December 2023. Patients’ demographic data were retrieved from patients’ files coupled with theatre record file, entered into a structured questionnaire, and then, in a statistical program.

**Results:**

A total of 290 patients underwent coronary artery bypass grafting at the center. The international missions performed a total of 159 (54.8%) operations, while the local team operated a total of 131 (45.2%) patients. The study showed significant statistical difference in terms of total operation time (95% confidence interval [CI] = 5.67, 6.01 vs. 95% CI = 6.32, 6.66), aortic cross-clamping time (95% CI = 75.92, 90.00 vs. 95% CI = 111.19, 126.65), and total cardiopulmonary bypass time (95% CI = 115.9, 134.75 vs. 95% CI = 174.52, 201.27) between the international missions and local surgical team, respectively. The mortality rate was higher in patients operated on by the local team (13.7%) than by international missions (8.8%), however there was no statistical difference.

**Conclusion:**

This study has shown the beneficial advantage of international surgical missions to newly established open-heart centers with advanced facilities and skill-deprived team. International surgical missions have greatly contributed to the progression of the center as they oversee and support the programs.

## INTRODUCTION

**Table:** 

Abbreviations, Acronyms & Symbols				
BMI	= Body mass index		EuroSCORE	= European System for Cardiac Operative Risk Evaluation
CABG	= Coronary artery bypass grafting		ICU	= Intensive care unit
CAD	= Coronary artery disease		JKCI	= Jakaya Kikwete Cardiac Institute
CI	= Confidence interval		LAD	= Left anterior descending artery
COVID-19	= Coronavirus disease 2019		NYHA	= New York Heart Association
CPBT	= Cardiopulmonary bypass time		OHI	= Open Heart International
DF	= Degrees of freedom		SD	= Standard deviation

The majority of low-income countries, especially of the sub-Saharan Africa, still have few centers for open-heart surgery programs^[1]^. Patients with cardiac diseases in these countries must be referred abroad for treatment or live in morbid conditions. Establishing open-heart surgery programs requires a governmental and collaborative well-organized and coordinated team. Despite all the challenges, there are a few centers in sub-Saharan Africa that have managed to conduct open-heart surgery as a result of a well-coordinated system set in place and backed by their governments. International missions to these centers have been crucial in provision of material support, organizing and helping to build up a core team that work together, and monitor their progression^[2]^. Besides all this, they also provide humanitarian surgical treatment to patients requiring open heart surgery. Despite the benefits provided by international missions to some centers, these have been observed with mixed results. The role of international cardiac surgical missions to newly established cardiac centers, especially of sub-Saharan Africa, has been received with different perceptions^[3]^. The missions usually have different targets - to provide humanitarian surgical treatment to patients coupled with improving skills of the local team and of fellowship students from their countries who accompany the missions. It appears that some of the international missions should be identified with great care because the primary goal is for the local surgical team to build their skills and eventually be able to handle their own patients with cardiac diseases. Ikechukwu A, from Nigeria, while reviewing the progress and success of open-heart surgery done by international missions concluded that missions were not able to provide adequate services for the care of congenital heart disease patients that required cardiac surgery in terms of reduction and provision of educational skills to the local team^[3]^. This study focus on coronary artery bypass grafting (CABG), which is one of the most advanced surgical procedures in cardiac surgery due to its technicality and is commonly and routinely done in developed countries^[4^-^6]^.

## METHODS

On May 21, 2008, open-heart surgery started to be performed in a single unit of the Department of General Surgery of Muhimbili National Hospital^[7]^. The team was only able to perform open-heart surgery in the presence of a visiting team from Zimbabwe, and by the first year, they were able to perform a total of 105 open-heart surgeries with an overall mortality of 13.4%^[7]^. Most of the cases that were operated on were congenital and chronic rheumatic valvular heart diseases; the team could hardly operate a double valve case. The visiting team continued to support the local team through 2012, then progressively declined the number of visits because the local team continued to acquire skills and competence - at that time, the local team was able to do single valve replacement and closing inborn defects. The Government, through its Ministry of Health and in collaboration with China, managed to establish and built up a Cardiac Center: a treatment and training center that by 2014, shifted into a new building and was made to be a solitary Department of Cardiovascular Medicine, still being a constituent of the Muhimbili National Hospital. By the year 2017, the Department was resolved, and a Cardiac Surgery Institute was established and renamed after the fourth president of the United Republic of Tanzania, The Jakaya Kikwete Cardiac Institute (JKCI), this name has gained popularity and remained to date. The institute has become a well-known cardiac center worldwide and was organized into six directorates among which a surgical directorate was established. The directorate of surgery formed three departments, namely Pediatric, Adult Cardiothoracic Surgery, and Vascular Surgery. The institute and the directorate of cardiac surgery have experienced a large number of surgical missions coming in the form of surgical camps that perform surgical procedures for 1-3 weeks’ time. These are Almutanda (from Saudi Arabia), Open Heart International (OHI) (from Australia), CardioStart (from the United States of America), Saifee (from Bombay, India), Albasam (from the Emirates), and more recently a single surgeon from Max Hospital (from New Delhi, India). These surgical missions have been visiting the institute for various periods in a year. They have been providing surgical services to patients and supervising the local team to acquire surgical skills.

Though open-heart surgery had started in May 2008, CABG was a dilemma, and most patients with coronary artery disease (CAD) were being referred abroad, especially to India, for treatment^[7]^. The surgical mission that used to visit the institute preferred to operate on pediatric patients rather than adult patients. But gradually with time, the team gained expertise and through government commitment to improve people’s health as a whole, the team was able to operate on adult patients including those with CAD. Saifee, OHI, and CardioStart missions were the forerunner for starting operating patients with chronic ischemic heart disease. At that time, the local team had done operations with at least one single surgeon from abroad. The local team gradually continued to gain expertise and skills from the visiting missions. Some of the missions visiting tried to look for a good training center for some staff from the institute. They were sent to train in India, Israel, the United States of America, and Brazil at different time intervals. To date, the JKCI has progressed well in terms of diagnostic service and skilled local staff. The center is currently operating on patients with coronary heart disease by doing on-pump CABG. The emergence of the pandemic Coronavirus disease 2019 (COVID-19) at the end of 2019, that restricted movement and travel from one country to another^[8^,^9]^, further strengthened the local team by identifying themselves that they have a key role to provide health care to its people. In turn, the people and the community as a whole had no other option but to be treated by the local team. For success of cardiac team and especially to newly established centers of the sub-Saharan Africa, government commitment should be a pre-requisite to such programs, there should be a local team trained in various subsections including critical care nurses, theatre nurses, biomedical engineers, and perfusionists apart from cardiac anesthesiologists and cardiac surgeons^[10^-^12]^. The government further strengthened and equipped the JKCI with modern and highly advanced diagnostic facilities.

This was a retrospective study that reviewed all patients who underwent coronary revascularization at JKCI from May 2016 to December 2023. The number of surgical procedures performed by different operating missions, their progress, and mortality were included. All patients who were operated on at JKCI after they have undergone coronary angiography and echocardiography and were finally diagnosed with CAD and for whom revascularization by interventional stent placement was not possible were enrolled into the study; those whose data could not be retrieved were excluded.

### Techniques

Induction of anesthesia was performed with full monitoring using electrocardiography, pulse oximetry, invasive arterial blood pressure, and central venous pressure monitoring. Opioids-based agents, either fentanyl 0.1 mg/kg with 0.05 mg/kg midazolam or 0.2 mg/kg etomidate, were administered. Rocuronium 1 mg/kg was typically administered for muscle relaxation. After endotracheal intubation, maintenance of anesthesia was achieved with a volatile anesthetic 1-2% sevoflurane end-tidal concentration. The left internal mammary artery was harvested in all patients and was used as a conduit to the left anterior descending artery. The left saphenous vein and/or the right saphenous vein were harvested from the lower limbs and used as conduit for the rest of other stenosed coronaries. There was no case where the right internal mammary artery was used. Patients who underwent on-pump CABG received continuous infusion of an opioid (fentanyl) and propofol during cardiopulmonary bypass. Patients were routinely cooled to 28-32°C depending on the complexity of the procedure. Blood cardioplegia was mixed into four parts of blood for one part of crystalloid cardioplegia. Among patients who underwent off-pump CABG, a tissue stabilizing forceps, Octopus, was used to stabilize the epicardium along the coronary artery, then distal anastomosis was done; on completion of the distal anastomosis, rewarming was initiated, and after the heart has attained sinus rhythm with adequate cardiac contractility and stable hemodynamics, a side-biting aortic clamp was applied on the proximal aorta, taking care if there was risk of atheroma, and proximal anastomosis was done. De-airing and hemostasis were done. Weaning was initiated by having the patient adequately rewarmed and arterial blood gases checked and corrected accordingly. Weaning was gradually done followed by reversal of anticoagulation using 3 mg/kg protamine sulphate, hemostasis was achieved through ligature and cautery. Usually, two to three chest drain tubes were inserted on the left pleurae, anterior mediastinum, and or the right pleurae whenever appeared to have been open. A temporary epicardial pacing wire was always inserted on the right ventricular free wall and exteriorized to the skin. The chest was closed using sternal wire in most of the cases, and whenever possible, an Ethibond® 5 suture was used to close the chest.

### Objectives

To determine and to compare the number of coronary artery revascularizations and mortality rates between the local team and international missions at the JKCI.

### Sample Size and Sampling

The sample size was 290 patients. A simple sampling procedure was used so that all patients who underwent coronary revascularization surgery were enrolled into the study.

### Ethical Consideration

Ethical clearance was obtained from the institutional review board of the JKCI with protocol number (AB:123/307/01K/13) and conducted in accordance with the Declaration of Helsinki.

### Data Collection

This was a retrospective study that reviewed all patients who were referred to the adult cardiac surgical department having undergone coronary angiography at JKCI or from any other hospital within the country whose surgical intervention was done at the center. Patient demographic data such as region of domicile, age, sex, weight, and height were entered into a structured data sheet. Patients’ clinical findings such as ejection fraction, presence of diastolic dysfunction, and comorbidities (such as diabetes mellitus and or hypertensive heart disease) were inquired; patients’ intraoperative findings, number of grafts, and aortic cross-clamping, total operation, and cardiopulmonary bypass times were collected. The postoperative total duration of ventilation and any presence of intensive care unit (ICU) complications were noted, and the total duration of ICU stay was determined. The patient was followed to the ward, the duration of hospital stay in the ward was determined, any long-term complications such as readmission, presence of wound infection, and whether or not death occurred were also followed up.

### Data Analysis

Data was entered into an IBM Corp. Released 2015, IBM SPSS Statistics for Windows, version 23.0, Armonk, NY: IBM Corp. program; frequency distribution and cross-tabulation were calculated. Categorical data were compared using Chi-square, independent Student’s *t*-test was used to compare mean for data with normal distribution. A *P*-value < 0.05 was considered to be significant.

## RESULTS

### Patients’ Demographic

There was a total of 290 patients with chronic ischemic heart disease who underwent CABG operation of whom 228 (78.6%) were male and 62 (21.4%) were female; male patients outnumbered female patients by 3.6 folds. The mean age was 63.9 ± 8.0 years. The majority of the study patients (87 [30%]) had no underlying comorbidities, however, isolated diabetes mellitus was found in 13.1%, isolated hypertensive heart disease in 25.5%, and hyperlipidemias in 12.1% of the patients. Both diabetes and hypertensive heart diseases were found in 10.3%, other comorbidities were infection with hepatitis B virus (0.7%), hypertensive heart disease with stroke (1%), chronic renal failure (1.4%), patient being seropositive for human immunodeficiency virus (1.4%), patient having undergone cardiac catheterization with or without percutaneous coronary intervention and followed by cardiac arrest (1%), gross regional wall motion abnormalities (1%) , patients with both diabetes and hypertensive heart disease plus chronic renal failure (1%), and age > 80 years (1%). The typical presenting symptoms were chest pain with mild, moderate, and severe pain in 11.4%, 22.1%, and 55.5%, respectively; however, in 11% of the patients, there was no typical angina pain. The majority of patients were in New York Heart Association (NYHA) class III (50.3%), followed by NYHA class II (37.6%).

### Preoperative Results

A total of 227 (78.3%) and 63 (21.7%) patients underwent on-pump and off-pump CABG, respectively. Off-pump CABG procedures were 59 (93.7%) cases, performed by the international missions, while the local team operated on four (6.3%) cases.

An independent sample *t*-test was performed on preoperative factors like mean age, body mass index, duration of symptoms, extent of chronic arterial disease, NYHA class, and European System for Cardiac Operative Risk Evaluation (EuroSCORE) II between the local team and international missions and found that there was no significant statistical difference (*P*>0.05). However, the left ventricular ejection fraction was found to be statistically significant (*P*=0.001) (Table 1).

**Table 1:** Preoperative parameters of local team and international missions.

Variables	Team	No. of Cases	Mean ± SD	*t*-test	DF	*P*-value
Age	Local	131	63.8 ± 7.7	0.89	288	0.929
	Mission	159	63.8 ± 8.4			
BMI	Local	131	27.6 ± 4.8	0.124	288	0.901
	Mission	159	27.7 ± 4.5			
Duration of symptoms	Local	131	13.0 ± 8.5	1.733	288	0.330
	Mission	159	11.4 ± 7.6			
Extent of CAD	Local	131	2.9 ± 0.3	0.121	288	0.725
	Mission	159	2.9 ± 0.3			
NYHA	Local	131	2.6 ± 0.8	0.154	288	0.877
	Mission	159	2.6 ± 0.7			
EuroSCORE II	Local	131	1.4 ± 0.4	0.4	288	0.888
	Mission	159	1.4 ± 0.4			
Left ventricular ejection fraction	Local	131	51.9 ± 11.7	3.762	288	0.001
	Mission	159	46.9 ± 10.7			

BMI=body mass index; CAD=coronary artery disease; DF=degrees of freedom; EuroSCORE=European System for Cardiac Operative Risk Evaluation; NYHA=New York Heart Association; SD=standard deviation

### Intraoperative Results

**Fig. 1:**
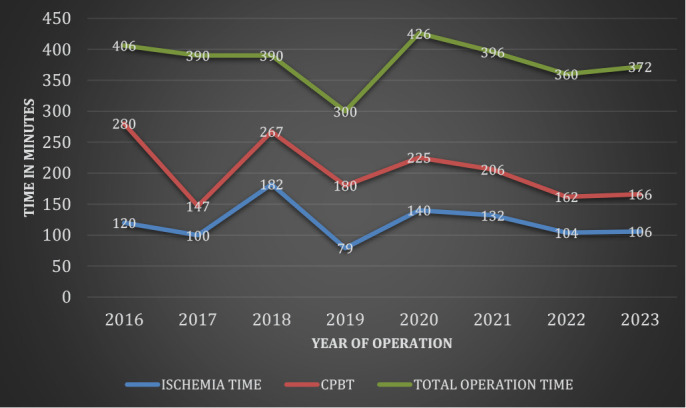
Mean values of aortic cross-clamping time, cardiopulmonary bypass time (CPBT) in minutes, and total operation time in hours, a trend over years.

An independent *t*-sample test was conducted to compare the intraoperative parameters such as number of grafts, duration of aortic cross-clamping, total duration of cardiopulmonary bypass time, total operation time, time interval to extubation, and total duration of ICU stay between the local surgical team and international missions, and it was found to have significant statistical difference (Table 2). A trend towards improved skills has been demonstrated in an overall reduction of the mean duration of aortic cross-clamping time, cardiopulmonary bypass time, and total operation time over the years (Figure 1).

**Table 2:** Intraoperative parameters compared.

Variables	Team	No. of Cases	Mean ± SD	*t*-test	DF	*P*-value
Number of grafts	Local	131	2.8 ± 0.6	2.459	288	0.015
	Missions	159	2.6 ± 0.7			
Duration of aortic cross-clamping	Local	127	118.9 ± 44	6.587	220	0.001
	Missions	95	83.0 ± 34.5			
Cardiopulmonary bypass time	Local	127	187.9 ± 76.2	7.086	220	0.001
	Missions	95	125.3 ± 46.3			
Total operation time	Local	131	6.4 ± 1	8.881	288	0.001
	Missions	159	5.4 ± 0.9			
Time interval to extubation	Local	131	20.0 ± 14.0	2.78	260	0.006
	Missions	159	16.24 ± 7.0			
Duration of ICU stay	Local	129	4.512 ± 1.838	2.397	285	0.017
	Missions	158	3.949 ± 2.084			

DF=degrees of freedom; ICU=intensive care unit; SD=standard deviation

### Postoperative Results

**Fig. 2:**
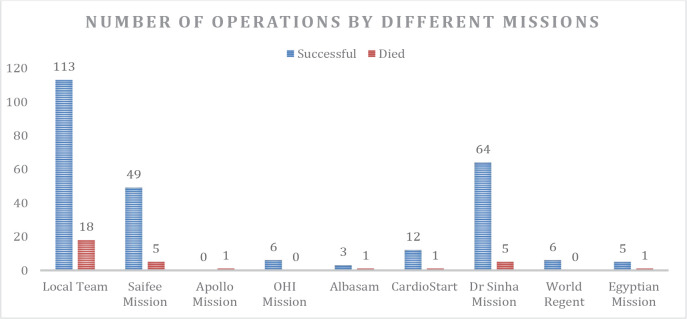
*Number of operations and corresponding number of deaths. OHI=Open Heart International.*

The local team operated on a total of 131 (45.2%) patients, while 159 (54.8%) patients were operated on by international surgical missions (Figure 2). Surgery was successful in 86.3% of the patients who were operated on by the local team, as compared to 91.2% of patients who were operated on by surgical missions. In both groups, the operability risk as assessed using the EuroSCORE II was equal - the majority of patients had low to moderate operability risk, 99.2% and 98.7%, for the local and international teams, respectively (Fishers’ exact test was 0.882, degrees of freedom [DF]=2, *P*=0.836). The mortality rate was higher in patients who were operated on by the local team, accounting for 13.7%, as compared to 8.8% of those who were operated on by the international missions, though this difference was not statistically significant.

In both teams, most of the patients had three grafts (194 [66.9%]), two grafts were preferred in off-pump CABG with the mission. In almost all patients who had a single graft, the operation was conducted with off-pump CABG.

**Fig. 3:**
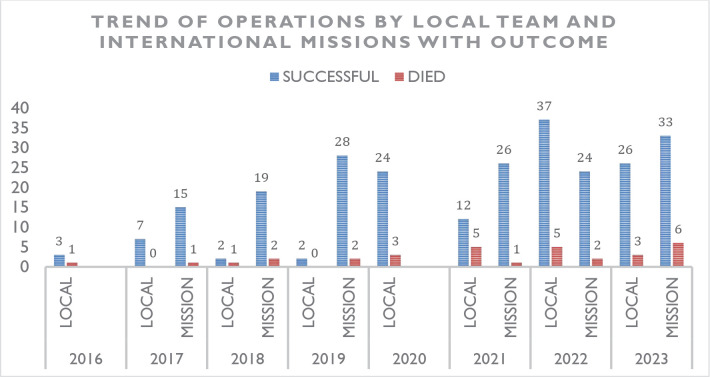
*Number of operations and corresponding deaths over the years, local team vs. international missions.*

The number of CABG progressively increased over the years, the local surgical team initially performed CABG at low output, from the year 2016 to 2019, but by the year 2020, there was a sharp increase in number of CABG performed by the local surgical team. During that time, there was a surge of patients reporting to the center diagnosed with chronic ischemic disease and there was also the COVID-19 pandemic, so no mission was coming from any country to conduct a surgical mission at the center. By the year 2021, the local team had gained adequate skills to perform CABG on their own and to date, the procedure is routinely done at the center (Figure 3).

The local surgical team was able to operate a total of 131 (45.2%) cases with 18 (13.7%) who demised, while the international missions as a whole were able to operate a total of 159 (54.8%) cases with a total of 14 (8.8%) patients who died.

Overall surgery was successful in 258 (89%) patients, mortality was found in 32 patients, giving an overall mortality rate of 11% among patients who underwent CABG (Table 3). There was a relatively higher mortality on patients who were operated on by the local team as compared to those operated on by international missions, 13.7% *vs.* 8.8%, respectively. However, there was no significant statistical difference (χ2 =1.782 [DF=1, N 290], *P*=0.126).

**Table 3:** Patient outcome from local team *vs.* international missions.

Outcome	Operating Teams		
	Local Team	International Missions	
Fully recovered	113 (86.6)	145 (91.2)	258 (89.0)
Died	18 (13.7)	14 (8.8)	32 (11.0)
Toral	131 (45.2)	159 (54.8)	290 (100.0)

## DISCUSSION

Surgical missions to countries with low or poor facilities for open-heart surgery are very crucial and important to these countries; apart from providing services, they train the local team and provide room for training to some of their institutions^[13]^. Aside from training and taking care of patients from patient selection to surgical intervention and critical care, they oversee the whole program in these countries and provide the spirit of working together as teamwork^[14^-^16]^.

The aim of this review was to determine and compare the number of coronary artery revascularizations and mortality rates between the local team and international missions at the center.

The review demonstrated that the operative outcomes following CABG in Africa are comparable to the results found in other regions. The large percentage of patients who underwent CABG were male^[17]^.

In a study conducted by Akintoye et al.^[18]^, the overall average cardiopulmonary bypass time was 112.98 minutes, which was lower than the one in our study - 187.9 minutes for the local team and 125.3 minutes for the international missions. The mean aortic cross-clamping time for the local team and international missions was 118.9 minutes and 83.0 minutes, respectively, which was longer compared to 67.3 minutes obtained from the same study^[18]^. This variation is because of the total number of operations done by teams and the difference in expertise between the two localities. The mortality rate from the local team and international missions were 13.7% and 8.8%, respectively, which were significantly higher than from a study done in Brazil by Paez et al.^[19]^ in 2019, with a sample size of over 2,000, having operative mortality of 2.8%. The overall mortality of 3.73% following CABG in Africa is due to lack of financial resources and poor infrastructure, hindering the optimal practice of CABG procedures^[18]^. The mortality seen in the international missions *vs.* local team showed difference due to the smaller number of expert surgeons capable of doing surgery at an accelerated rate in East Africa.

### Limitations

The study was limited by the inability to report the overall causes of mortality in both local team and international missions. Additionally, the study did not account for the rates of in-hospital complication rates, which resulted to significant morbidity in the patients. As a retrospective study, which was conducted over a certain time frame, the study did not take into account changes in medical practices, conduct of surgery, and patient populations. The study did not track long-term outcomes or follow-up after hospital discharge, which meant that the full scope of patient recovery or emergence of late complications were not assessed.

## CONCLUSION

This study has shown the beneficial advantage of international surgical missions to newly established open-heart centers, especially those with advanced facilities and skill-deprived team. The skills acquired by the local team through surgical missions enabled the local team to handle their own patients especially during the period of the COVID-19 pandemic and continued to perform surgical operation at a higher patient output with minimal mortality rate. The number of skilled teams has dramatically increased coupled with good patient outcome.

## References

[1] Manuel V, Miana LA, Edwin F. (2021). Narrative review in pediatric and congenital heart surgery in
sub-Saharan Africa: challenges and opportunities in a new
era. AME Surg J.

[2] Santos CA, Oliveira MA, Brandi AC, Botelho PH, Brandi Jde C, Santos MA (2014). Risk factors for mortality of patients undergoing coronary artery
bypass graft surgery. Rev Bras Cir Cardiovasc.

[3] Ke O, Bode F, Yb A, Aj O, Mb A, Uu O. (2021). Surgical 'safari' vs. educational program: experience with
international cardiac surgery missions in Nigeria - a
rejoinder. Braz J Cardiovasc Surg.

[4] Fenton KN, Castillo SH, Claro CD, Novick WM. (2011). Teamwork and program organization in developing
countries. World J Pediatr Congenit Heart Surg.

[5] Tiwari KK, Grapsa J, Laudari S, Pazdernik M, Vervoort D. (2021). Challenges and possibilities of developing cardiac surgery in a
peripheral hospital of lowand middle-income countries. Perfusion.

[6] Oludara MA, Nwiloh J, Fabamwo A, Adebola P. (2014). Commencing open heart surgery in resource limited countries:
lessons from the LASUTH experience. Pan Afr Med J.

[7] Nyawawa E, Ussiri E, Chillo P, Waane T, Lugazia E, Mpoki U (2010). Cardiac surgery: one year experience of cardiac surgery at
Muhimbili national hospital, Dar es Salaam- TANZANIA. East Cent Afr J Surg.

[8] Tarimo CS, Wu J. (2020). The first confirmed case of COVID-19 in Tanzania: recommendations
based on lesson learned from China. Trop Med Health.

[9] Cucinotta D, Vanelli M. (2020). WHO declares COVID-19 a pandemic. Acta Biomed.

[10] Vervoort D, Swain JD, Pezzella AT, Kpodonu J. (2021). Cardiac surgery in lowand middle-income countries: a
state-of-the-art review. Ann Thorac Surg.

[11] Ancona C, Agabiti N, Forastiere F, Arcà M, Fusco D, Ferro S (2000). Coronary artery bypass graft surgery: socioeconomic inequalities
in access and in 30 day mortality. A population-based study in Rome,
Italy. J Epidemiol Community Health.

[12] Yu TH, Hou YC, Chung KP. (2014). Do low-income coronary artery bypass surgery patients have equal
opportunity to access excellent quality of care and enjoy good outcome in
Taiwan?. Int J Equity Health.

[13] Reiche S, Mpanya D, Vanderdonck K, Mogaladi S, Motshabi-Chakane P, Tsabedze N. (2021). Perioperative outcomes of coronary artery bypass graft surgery in
Johannesburg, South Africa. J Cardiothorac Surg.

[14] Novokreshchenova IG, Senchenco IK. (2015). Quality of medical care for the elderly in out-patient conditions
according to the sociological survey. Russian Open Medical Journal.

[15] Ghosh P. (2005). Setting up an open heart surgical program in a developing
country. Asian Cardiovasc Thorac Ann.

[16] Mocumbi AO. (2012). Lack of focus on cardiovascular disease in sub-Saharan
Africa. Cardiovasc Diagn Ther.

[17] Adelborg K, Horváth-Puhó E, Schmidt M, Munch T, Pedersen L, Nielsen PH (2017). Thirty-year mortality after coronary artery bypass graft surgery:
a Danish nationwide population-based cohort study. Circ Cardiovasc Qual Outcomes.

[18] Akintoye OO, Fasina OP, Adiat TS, Nwosu PU, Olubodun MO, Adu BG. (2023). Outcomes of coronary artery bypass graft surgery in Africa: a
systematic review and meta-analysis. Cureus.

[19] Paez RP, Hossne NA, Santo J, Berwanger O, Santos R, Kalil R (2019). Coronary artery bypass surgery in Brazil: analysis of the
national reality through the BYPASS registry. Braz J Cardiovasc Surg.

